# Detecting and addressing eating disorders among individuals experiencing food insecurity: considerations for dietetic practice

**DOI:** 10.3389/fnut.2025.1668349

**Published:** 2025-09-12

**Authors:** Heather A. Davis, Emily Myers, Elena Serrano, Sarah Misyak

**Affiliations:** ^1^Department of Psychology, Virginia Tech, Blacksburg, VA, United States; ^2^Department of Human Nutrition, Food, and Exercise, Virginia Tech, Blacksburg, VA, United States

**Keywords:** eating disorders, food insecurity, stigma, RDN, weight neutral

## Abstract

In the United States, almost 10% of Americans will experience an eating disorder in their lifetime. Despite evidence that eating disorders occur across socio-economic backgrounds, the stereotypes of eating disorders being a disease of affluence persist. The experience of food insecurity, defined as limited or inconsistent physical and economic access to a sufficient amount and variety of nutritious food needed for a healthy life, is significantly associated with greater eating disorder symptoms. There are several reasons eating disorder symptoms may develop in people experiencing food insecurity, including food/benefit distribution cycles, shame, and weight bias. This Perspective highlights the relationship between food insecurity and eating disorders and provides informed recommendations specific to dietetic practice. Guidance is provided for Registered Dietitian Nutritionists (RDNs) in settings that serve individuals at risk of, or experiencing, food insecurity. RDNs should be informed on best practices for screening for eating disorders and providing appropriate referrals to eating disorder specific care, as well as encouraging realistic, achievable health behaviors, and using non-stigmatizing language.

## Introduction

In the United States, an estimated 9%, or 28.8 million, of Americans will experience an eating disorder in their lifetime (1). Eating disorders are mental health conditions characterized by disturbances in eating patterns and body image that arise from a combination of biological, psychological, and social factors. Despite evidence that eating disorders occur across socio-economic backgrounds, stereotypes of eating disorders being a disease of affluence persist (2, 3).

The experience of food insecurity, defined as limited or inconsistent physical and economic access to a sufficient amount and variety of nutritious food needed for a healthy life, is significantly associated with greater eating disorder symptoms, including dietary restriction not due to food scarcity, extreme weight control behaviors, and binge eating, and eating disorder diagnoses, such as bulimia nervosa and binge eating disorder (4–11). Compared to individuals with adequate food access, individuals experiencing food insecurity are 3–5 times more likely to also experience an eating disorder (7). Strikingly, this association remains present even when controlling for relevant socio-demographic factors such as age, gender, race, income, and education, highlighting the strong relationship between food insecurity and eating disorders (7, 12, 13). A summary of a sample of studies of eating disorders among individuals experiencing food insecurity is presented in the top panel of Table 1.

Table 1Summary of key studies (top panel) and theoretical frameworks (bottom panel) describing the relation between eating disorders and food insecurity.

**Table:** 

**Key studies**			
**Study**	**Sample/population**	**Study design**	**Key findings**
Abene et al. (14)	Meta-analysis of adults from 13 articles	Cross-sectional	Adults with food insecurity were 1.66 times more likely to have binge eating and 2.70 more likely to have binge eating disorder compared to adults without food insecurity.
Barry et al. (8)	Children aged 8-10 years from households with incomes ≤ 200% of the federal poverty line in southeastern Michigan, U.S. (*N* = 194)	Cross-sectional	Children with food insecurity had higher average Children's Eating Attitudes Test total scores relative to children without food insecurity. Child food insecurity was also associated with greater preoccupation with food and weight, and more social pressure to eat relative to children with food security.
Becker et al. (13)	Adults (*N* = 503) that visited U.S. food pantries	Cross-sectional	Twelve percent of participants with individual-level food insecurity reported clinically significant ED pathology. Sixteen percent of participants in the highest category of food insecurity (household with child hunger) reported clinically significant ED pathology. Compared with participants with lower levels of food insecurity, higher food insecurity was associated with higher levels of binge eating, overall eating disorder psychopathology, compensatory behaviors, and dietary restraint for any reason.
Christensen et al. (11)	U.S. college students (*N* = 579)	Cross-sectional	The prevalence of probable eating disorder diagnosis among college students with food insecurity was 47.6% as compared to 31.3% among college students without food insecurity. The most common probable diagnoses were bulimia nervosa and other specified feeding and eating disorder.
Kosmas et al. (44)	U.S. college students (*N* = 259)	Longitudinal across 3 months	At 3-month follow-up, baseline food insecurity significantly predicted greater cognitive restraint, dietary restriction, excessive exercise, and purging, adjusting for baseline levels, sociodemographic characteristics, and body mass index. Baseline food insecurity did not predict binge eating at 3-month follow-up.
Hazzard et al. (7)	U.S. adults, nationally representative (*N* = 2,914)	Cross-sectional	Participants with food insecurity were 4.8 times more likely to be diagnosed with bulimia nervosa and 3.49 times more likely to be diagnosed with binge-eating disorder.
Hazzard et al. (17)	Teenagers followed through young adulthood (*N* = 1,813)	Longitudinal across 10 years	Cross-sectionally, severe FI was significantly associated with greater prevalence of all eating disorder behaviors, with the strongest associations being with binge eating and extreme weight control behaviors. Longitudinally, severe food insecurity associated with greater binge eating five years later after accounting for prior binge eating.
Hazzard et al. (18)	Young adults in the U.S. who had experienced past-month household food insecurity (*N* = 75)	14-day ecological momentary assessment, four surveys per day	Momentary greater food security relative to one's typical level of food security predicted greater subsequent binge eating. The association was present only for those who used food assistance programs, reported high engagement in resource trade-off coping strategies (e.g., skipping payment of other expenses to purchase food), or had low self-efficacy related to food security.
Hazzard et al. (19)	Racially and ethnically diverse parents (*N* = 1120)	Longitudinal across 18 months	Household food insecurity was cross-sectionally and longitudinally (across 18 months) associated with greater weight control behaviors and binge eating. Food insecurity significantly predicted the onset of compensatory weight control behaviors at follow-up only among those participating in federal food assistance programs.
Hooper et al. (9)	*N* = 559 midlife and older adult food bank clients [*N* = 292 midlife (51–65 years) and *N* = 267 older adults (66+ years)]	Cross-sectional	Of the total sample, 8.9% of participants had a likely eating disorder: 10.5% of midlife adults, 5.6% of older adults. Among midlife adults, the most prevalent symptom was binge eating. Food insecurity severity was associated with greater night eating, binge eating, skipping ≥two meals in a row, and laxative use. The same associations were significant for older adults, with the addition of vomiting and exception of laxative use.
Nagata et al. (10)	National cohort of 9- to 14-year-old children (*N* = 10,035)	Longitudinal across 2 years	Food insecurity at baseline was associated with 1.31 higher odds of binge eating and 1.67 higher odds of clinical threshold binge eating disorder or subthreshold binge eating disorder two years later.
Zickgraf et al. (12)	U.S. college students, (*N* = 121,627)	Cross-sectional	Food insecurity was significantly associated with 1.19 times greater likelihood of an eating disorder when adjusting for concurrent anxiety and depression.

**Table:** 

**Theoretical frameworks to explain how and why eating disorders develop among individuals experiencing food insecurity**		
**Theoretical framework**	**Brief description**	**Supporting studies**
The feast-or-famine cycle	The feast-or-famine cycle may underly the robust relation between food insecurity and binge eating. Food intake is thought to increase during periods of relative food abundance (such as following the receipt of federal food assistance benefits) and decrease during periods of food scarcity. The inconsistency in the availability and consumption of food triggers feelings of deprivation which increase vulnerability to binge eating.	Goode et al. (24), Hazzard et al. (17–19)
Shame	Feelings of shame, due to finances and food access, are common among individuals experiencing food insecurity. Shame is also a robust risk factor for eating disorder symptoms across studies. As such, shame may be a mechanism underlying the relation between food insecurity and eating disorder symptoms.	Davis et al. (30)
Weight bias and discrimination	Weight bias and discrimination occur prevalently in healthcare settings. Because food insecurity is associated with unintentional weight gain and high body weight, individuals experiencing food insecurity may have a higher likelihood of experiencing weight bias and discrimination. In turn, these experiences of bias and discrimination may lead to stress or pressure to lose weight that then increases risk for engagement in extreme weight control behaviors characteristic of an eating disorder. Longitudinal evidence supports that food insecurity precedes the development of eating disorder behaviors; weight bias and discrimination may thus be an underlying mechanism of this relation.	Briggs et al. (46), Davis et al. (47), Kosmas et al. (44), Taylor et al. (45)

When working with clients with food insecurity, Registered Dietitian Nutritionists (RDNs) are uniquely positioned to assess eating disorder symptoms and develop individualized eating and nutrition recommendations. Considering the intersection between eating disorder symptoms and food insecurity, special attention is warranted to mutually address both concerns with each unique individual, while not exacerbating eating disorder symptoms.

The aim of this Perspective is to provide guidance to dietetic practitioners in settings that serve individuals at risk of, or experiencing, food insecurity. We first summarize the literature on the overlap between food insecurity and eating disorders. We then describe foundational theories underlying the development of eating disorder symptoms in the context of experiencing or having previously experienced food insecurity. We then offer theory- and evidence-informed recommendations for dietitians. Finally, we provide recommendations to help ensure that nutrition interventions do not inadvertently contribute to the development or exacerbation of eating disorders among individuals experiencing or who have previously experienced food insecurity.

## How and why do eating disorder symptoms develop among individuals experiencing food insecurity?

Several hypotheses exist for how and why eating disorder symptoms develop in the context of food insecurity, including food distribution cycles, shame, and weight bias. These factors can present independently or in conjunction with one another.

### The feast-or-famine cycle

Of the many eating disorder symptoms, there is a particularly robust relationship between food insecurity and binge eating, defined as consuming a large amount of food while experiencing a loss of control (14). One prevailing theory for the development of binge eating within food insecurity is the “feast or famine cycle”, whereby food intake increases during periods of relative food abundance and decreases when food again becomes scarce (15, 16). Inconsistency in the availability and consumption of food, regardless of the reason for food restriction (e.g., due to a desire for weight loss/maintenance or due to food scarcity), is thought to trigger feelings of deprivation, predict preoccupation with food, and increase vulnerability to subsequent binge eating (17–20). Having autonomy and agency to choose foods to purchase and consume is associated with greater food satisfaction and eating behavior regulation (21). However, experiencing food insecurity makes it difficult to have autonomy in food choices, given limited access to sufficient quantity and variety of foods, and the limitations on types of foods that can be purchased with some federal nutrition assistance benefits (22). This lack of autonomy may be a contributor to feelings of deprivation that lead to binge eating among individuals experiencing food insecurity—research should evaluate this possibility.

Food distribution frequency may also contribute to the “feast or famine” phenomena and serve as a factor, notably monthly benefit distribution schedules of nutrition assistance, such as Supplemental Nutrition Assistance Program (SNAP) benefits. This consistent yet infrequent disbursement may unintentionally facilitate the feast or famine cycle for some recipients, compared to staggered distributions (23). A 2023 study demonstrated that relative food abundance at one time point predicted later engagement in binge eating (18). This relationship was strongest among individuals reporting use of food assistance programs. An additional study showed that experiences of loss of control and emotional eating occurred frequently upon disbursement of benefits when food supply was high, and also occasionally when resources were diminishing, with some participants noting that worrying about food access resulted in overeating (24). The cyclical nature of food availability may play a role in the onset and maintenance of binge eating. Importantly, although prior work has included both longitudinal and cross-sectional designs, more work using longitudinal designs (across short and extended time periods) and experimental methods to demonstrate temporal and causal effects is needed.

### Shame

Many individuals experiencing food insecurity reported feeling ashamed of their financial limitations (25, 26). Shame is a negative emotion that occurs as a result of feeling morally inferior to others (27). Shame about finances and food access may translate to attitudes toward eating. For example, individuals receiving SNAP benefits have described a sense of morality around needing to make “good food choices” (24). Additionally, they reported feeling pressure to engage in extreme behaviors to lose weight (24). Importantly, shame is a robust predictor of eating disorder symptoms, including binge eating, purging, and excessive exercise (28, 29). Women experiencing food insecurity and an eating disorder or other mental health disorder have reported higher levels of shame than women experiencing food insecurity without a mental health disorder (30). As such, shame may be a mechanism underlying the relationship between food insecurity and eating disorder symptoms. Of note, prior work on shame, eating disorders, and food insecurity has been cross-sectional; longitudinal and experimental designs are needed to support the potential temporal and causal effects of food insecurity on shame and subsequent eating disorder symptoms.

### Weight bias and discrimination

Weight bias and discrimination may also be contributors to eating disorders among individuals experiencing food insecurity. Weight bias is defined as generalized negative beliefs about people of high body weight (31). Weight discrimination is defined as poor treatment of an individual due to their body size (32). Unfortunately, weight bias and discrimination are often viewed as socially acceptable forms of discrimination in our society (33, 34). Weight bias in particular is prevalent in healthcare settings—including among dietitians (35, 36)—and is associated with less healthcare utilization and worse health outcomes among people of higher body weight (31). Although most practitioners aim to avoid discrimination, implicit biases—unconscious attitudes or stereotypes—can sometimes lead to unintentional stigmatization. Because stigmatization for any reason can result in stress that leads to or exacerbates maladaptive coping behaviors, such as eating disorder symptoms, it is crucial that practitioners take steps to recognize their own implicit biases to avoid unintentional stigmatization and to support clients whose eating behavior might be influenced by external sources of bias and discrimination.

The presence of food insecurity is associated with unintentional weight gain and high body weight (6, 37), resulting in a higher likelihood of experiencing weight bias and weight discrimination. Research has identified low diet quality, including lower consumption of nutrient-dense foods and higher consumption of energy-dense and highly processed foods, as a strong contributor (38). This discrepancy can be partially attributed to greater availability and affordability of ultraprocessed and energy-dense foods in low-income vs. high-income areas (39, 40). Concerningly, weight discrimination is a powerful social determinant of health (33). Across studies, the experience of weight discrimination and internalization of weight bias among individuals with food insecurity is associated with a higher number of eating disorder symptoms, lower quality of life, and psychosocial impairment (41–43).

The experience of weight bias and discrimination, in conjunction with higher weight, among individuals experiencing food insecurity may lead to engagement in inappropriate weight control behaviors. For example, the experience of food insecurity predicts later engagement in self-induced vomiting, excessive exercise, and dietary restriction not due to food scarcity (19, 44). Individuals experiencing food insecurity and weight-based discrimination report finding the experience stressful and using dietary restriction to mitigate weight gain (45, 46). Finally, weight bias predicts eating disorder diagnosis among women experiencing current food insecurity, even after adjusting for current weight (47), suggesting that weight bias is robustly associated with eating disorder pathology regardless of weight status. Notably, research in this area has been largely cross-sectional and self-report survey-based; future research should use longitudinal or experimental designs to verify the potential causal effects of this model.

A summary of these frameworks and supporting studies is presented in the bottom panel of Table 1.

## Recommendations for dietetic practice

### Eating disorder screening

Because individuals with food insecurity are more likely to experience an eating disorder than individuals without food insecurity, RDNs should ideally screen individuals who are currently experiencing or have experienced food insecurity for eating disorders or eating disorder symptoms. Based on research on the feast or famine cycle (18, 24), this recommendation is particularly pertinent for individuals currently receiving nutrition assistance benefits. A recommended screening measure is the SCOFF (48), which is widely used, brief, and demonstrates good sensitivity and specificity in detecting eating disorders, including among individuals experiencing food insecurity (49, 50). The SCOFF includes five items that assess whether an individual (1) engages in self-induced vomiting, (2) has concern about losing control while eating, (3) has experienced recent weight loss, (4) perceives themselves as “too fat”, and (5) experiences food as “dominating” their life. The SCOFF and other common eating disorder screening and diagnostic tools are summarized in Table 2 (51–56). In addition to screening and diagnostic measures, RDNs can ask their clients open-ended questions regarding how food insecurity may influence the individual's eating behavior, weight, and exercise routine. Because eating disorder assessments were not developed with food insecurity considerations in mind (39), it is possible that clients could have trouble answering some questions. For example, they may eat small meals for reasons that vary, perhaps due to lack of access to food and other times due to eating disorder-related thought patterns. Correspondingly, recommendations to increase meal size should be tailored to reflect the reason(s) for limiting size. To allow for comprehensive assessment, open-ended and semi-structured questions are needed in addition to screening instruments such as the SCOFF. See Table 3 for examples of preliminary questions RDNs may ask clients following the administration of screening instruments. We note that the preliminary example questions presented in the Table 3 were generated by our research team (i.e., a clinical psychologist, RDN, Assistant Director and Director of the Virginia Cooperative Extension Family Nutrition Program). Importantly, the preliminary questions have not yet been systematically investigated or piloted in any practitioner or client populations. We recommend that future research investigate these and other culturally sensitive questions to assess eating patterns and eating disorder symptoms among individuals experiencing food insecurity to ensure that they yield the information intended.

**Table 2:** Commonly used self-report screening and diagnostic tools for eating disorders.

**Tool**	**Number of items**	**Purpose**	**Description**
SCOFF (48)	5	Screening	Evaluates the presence of the following eating disorder symptoms: self-induced vomiting, loss of control eating, weight loss, feeling fat, and perceived centrality of food to life. Participants indicate “yes” (scored 1) or “no” (scored 0) to each item. Scores ≥2 indicate a probable eating disorder. The measure has high sensitivity and specificity across studies, including among individuals experiencing food insecurity (49, 50).
Binge Eating Disorder Screener (BEDS-7) (51)	7	Screening	Assesses for overeating in the last 3 months. If clients answer yes to overeating, six additional questions consistent with the criteria for DSM-5 binge eating disorder are administered, including presence of loss of control, guilt about eating, embarrassment, and self-induced vomiting following binge eating (for differentiation from bulimia nervosa). The BEDS-7 demonstrates 100% sensitivity and 38.7% specificity for detecting binge eating disorder. (51)It has not yet been tested among individuals experiencing food insecurity.
Eating Disorder Diagnostic Scale (EDDS) (55)	23	Diagnosis	Used to diagnose DSM-5 eating disorders. Items assess eating disorder symptoms and behaviors over the past 3-6 months. The EDDS demonstrates convergent validity, test–retest reliability, and internal consistency (52, 53). Among individuals experiencing food insecurity, items assessing eating large amounts of food when not physically hungry and feeling negatively about overeating demonstrate poor ability to differentiate between different levels of eating disorder pathology (54).
Eating Disorder Examination Questionnaire (EDE-Q) (55)	28	Diagnosis	Assesses DSM-5 eating disorder symptoms and pathology over the past four weeks. A global score of overall eating pathology is calculated from 22 items. The global score consists of four subscales: Restraint, Eating Concerns, Shape Concerns, and Weight Concerns. Items are rated on a 7- point Likert scale anchored at 0 = “Never,” and 6 = “All of the time/Every day”. A systematic review showed strong support for psychometric properties (56). Among individuals with food insecurity, items assessing (1) eating large amounts of food and (2) negative emotions about overeating are *more* likely to be endorsed with low eating disorder pathology but *less* likely to be endorsed with high eating disorder pathology (54).

**Table 3:** Preliminary questions that may be used to further assess eating disorder behaviors and cognitions in individuals experiencing food insecurity.

**Topic**	**Questions**
Eating behaviors	How often do you skip meals? When you skip meals, how often is it because there isn't enough food versus other reasons? [If there are other reasons]: What are some other reasons you don't eat? How do you decide what and how much to eat when food is limited? When you're able to access more food, do you ever still feel the need to limit how much you eat? [If yes:] Tell me about that. How have your thoughts or feelings about eating changed since having difficulty getting enough food? Are there any rules you feel you need to follow around food? [If yes:] Tell me about them. What's it like for you emotionally when food becomes more or less available? Do you ever eat more than you plan to? [If yes:] What is that like for you? How do you feel? Do you ever try to make up for eating in any way, like skipping meals later, exercising more, or other methods? [If yes:] What do you do? Have you ever felt ashamed or guilty about the way you eat? [If yes:] Tell me about that. Has anyone ever said anything about your eating habits that has stuck with you? [If yes:] Tell me about it.
Body image	How do you feel about your body size and shape? How have your thoughts or feelings about your body size changed since you started having difficulty getting enough food? How do your thoughts and feelings about body size relate to difficulty getting enough food? Have you ever felt uncomfortable in your body because of how you're eating patterns change when food is scarce or unpredictable? [If yes:] Tell me about that. Are there messages you've heard—maybe from family, media, or doctors—that have stuck with you about your body size? [If yes:] Tell me about them. Do you ever feel judged—for your body, eating habits, or health? Do you ever avoid eating because of how you feel about your body or weight? If yes: Tell me about that.

These preliminary example questions were generated by our research team (i.e., a clinical psychologist, RDN, Assistant Director and Director of the Virginia Cooperative Extension Family Nutrition Program). Importantly, the preliminary questions have not yet been systematically investigated or piloted in any practitioner or client populations.

### Referrals to eating disorder-specific care

When eating disorder symptoms are detected, individuals should be referred to eating disorder-specific mental health care that can be provided simultaneously with medical nutrition therapy. A comprehensive treatment team for addressing eating disorders includes a therapist, dietitian, and physician, as well as possible adjunctive care. Referrals may be sourced from the National Eating Disorders Association Provider Finder, a database of therapists who specialize in eating disorders (57). Fortunately, access to eating disorder specialists via telehealth has expanded access to treatment in more rural settings and for those with more limited access to healthcare (58). Unfortunately, in some countries, eating disorder and mental health care is not universally covered by health insurance, and individuals experiencing food insecurity may be un- or under-insured (59). In these cases, it may be difficult to find affordable eating disorder and mental health care (59). Digital, self-guided interventions are low-cost, low-burden, and can be accessible via Smartphone or computer (60). As such, digital interventions offered alongside medical nutrition counseling or within programs that target individuals who are at risk of food insecurity, such as the Supplemental Nutrition Assistance Program—Education (SNAP-Ed) and the Expanded Food and Nutrition Education Program (EFNEP) offer promising alternatives to traditional eating disorder and mental healthcare. Future research should investigate the implementation and outcomes of such interventions.

### Encouraging realistic and achievable health behaviors

Practitioners should be cautious about prescribing health behaviors and recommending weight loss in the context of food insecurity. If individuals feel pressure to lose weight, particularly from health professionals, but have limited access and autonomy around food, they may resort to extreme measures, such as extreme dietary restriction, to manage weight (45, 46). Because dietary restriction increases risk for binge eating, it is unlikely to yield weight loss (61). Practitioners should discuss with their clients the harms of using extreme weight control methods and emphasize health behavior engagement rather than weight outcomes. A weight-neutral approach focused on actionable health behaviors and outcomes rather than a weight-centric approach focused on weight outcomes can encourage clients to prioritize their health while maintaining a positive relationship with food and their bodies. A 2019 review suggests that weight-neutral approaches for health may be as effective as traditional weight-loss methods for improving physical, psychological, and behavioral outcomes (62). For example, practitioners should educate clients on eating as consistently as possible to reduce binge eating vulnerability, while also recognizing that this may be challenging for individuals experiencing food insecurity. Practitioners may also educate individuals on the health benefits of engaging in regular movement that does not require a great deal of resources or time (e.g., walking, yoga at home) to increase mobility and heart health, avoiding cigarette smoking, limiting alcohol use, and prioritizing healthy sleep practices (e.g., sleeping 6–8 h/night). Each of these recommendations lessens pressure on diet quality and weight-related outcomes and increases emphasis on caring for oneself, which may prevent or reduce pathological eating and weight control behaviors as well as risk for other chronic health problems (63, 64).

### Practicing compassion and using non-stigmatizing language

Unbiased and compassionate care is especially important for clients experiencing food insecurity, given potential high levels of shame, which increases vulnerability to eating disorders (25, 26, 30). Frequent compassionate responses that express care, non-judgment, and validation of the individual's specific challenges are recommended. Relatedly, it is also crucial to recognize that individuals experiencing food insecurity are often subject to a myriad of stereotypes related to finances, health, and weight, all of which can interfere with their access to resources and perceived ability to change their behaviors (45). Recommendations related to reducing or maintaining weight may be intended to help enhance a patient's health, but such a recommendation could inflict harm rather than improve health. Practitioners should avoid encouraging changes in a client's weight unless it is markedly impacting their health. Even so, prescribing weight loss to manage health conditions, such as heart disease, non-alcohol fatty liver disease, or joint pain, may be counterproductive, given that achieving and sustaining weight loss overtime is statistically unlikely (65–67). A BMI of 25–30 is associated with the lowest mortality rates of all weight categories, suggesting that addressing weight with clients in this BMI range may not be necessary (68, 69). A focus on weight may even be counterproductive as weight cycling—losing and gaining weight frequently—itself is associated with negative health outcomes (70–72). Further, multiple large-scale randomized clinical trials indicate that decreases in disease incidence can occur with improved diet quality (e.g., greater consumption of fruits, vegetables, whole grains, etc.) and physical activity among individuals of all sizes, whether or not weight change occurs in the process (73–76). This suggests that compassionate encouragement of health behaviors without an emphasis on weight is likely to enhance health. Employing a weight-neutral approach with all clients, but particularly those experiencing food insecurity, can encourage clients to practice health behaviors without triggering disordered eating patterns. An example of a case vignette is presented in Table 4.

**Table 4:** Case Vignette of a patient experiencing food insecurity and eating disorder symptoms.

Jessica^*^ is a 35-year-old single mother of three children who receives SNAP benefits for her family at the usual disbursement of one time per month. Jessica screened positive for a possible eating disorder on the SCOFF. She described to her RDN that she recently unintentionally gained weight, which she attributed to overeating and relying on high calorie, processed foods at the end of the month when her SNAP benefits have run out. She explained that her overeating episodes often occur at the beginning of the benefit disbursement period, when she first receives her benefits. However, she stated that even though her benefits allow her to buy enough food to feed her household for the first two weeks of the month, she still attempts to restrict her food intake throughout the day to ensure that her children are getting enough to eat during that time period. Jessica reported that as a result, she becomes very hungry at night and when food is available, she overeats and experiences a loss of control over her eating. During the most recent episode, she reported eating six slices of frozen pizza. She stated that she experienced feelings of shame and guilt afterward due to concerns about eating “too much”, not leaving any for her children, and gaining more weight. She reported that she attempted to not eat for the entire following day to compensate for this episode. Jessica described feeling worried about her weight gain because she recently did not like how she looked in her clothing and her primary care physician had indicated the weight gain was Jessica's fault and made several comments about her needing to eat healthier and lose weight at her last appointment.
Jessica's RDN validated Jessica's distress and noted that her body's psychological and physiological response to the inconsistent availability of food was understandable given her circumstances. The RDN expressed compassion for the shame Jessica felt at being lectured by her primary care physician. Jessica's RDN then provided her with psychoeducation on the restriction and binge eating cycle. The RDN encouraged Jessica to eat a meal or snack every three hours to disrupt this cycle and prevent her from becoming overly hungry and vulnerable to binge eating. When Jessica expressed concern about not having enough “healthy” food available to eat every three hours, her RDN encouraged her to prioritize consistent nourishment over perfect nutrition, as eating consistently would help to stabilize her blood sugar, support hormonal balance and appetite regulation, and build a healthier psychological relationship with food and eating. The RDN provided Jessica with examples of low-cost and nutritionally dense foods that she could eat during periods of food scarcity (e.g., peanut butter, oatmeal, canned fish). In addition, the RDN was sure to also normalize and de-stigmatize Jessica's consumption of processed or convenience foods when they are all that is available to her. Finally, the RDN provided Jessica with a referral to a community psychology clinic that offered services on a sliding fee scale so that Jessica could work with a psychotherapist to further address her binge eating, body image concerns, and experiences of weight stigma.

* Pseudonym, identifying details have been changed to protect the privacy of the individual for whom this case vignette is based upon.

## International parallels and considerations

Although this Perspective focuses primarily on United States-based nutrition assistance programs (e.g., SNAP, EFNEP), the parallels described exist internationally. For example, Canada's Prenatal Nutrition Program (77), the United Kingdom's Healthy Start vouchers (78), and Brazil's Bolsa Família Program (79) each include some form and combination of food, financial support and nutrition education, and RDNs are often involved, at some level, in these efforts. Such programs exist because food insecurity is an urgent international concern: nearly 2.4 billion people, equivalent to 29.6 percent of the global population, experience food insecurity (80). Importantly, meta-analyses, systematic reviews, and recent empirical studies indicate that the association between food insecurity and eating disorders is present across countries and cultures (14, 81, 82).

Notably, access to treatment for eating disorders varies significantly depending on the structure of a country's healthcare system (83, 84). Universal healthcare, as provided in the United Kingdom and Australia, for example, provides more consistent access to not only general and primary medical care but also mental health services, which may contribute to more timely detection and treatment of eating disorders regardless of food security status (85). However, despite greater coverage of services, barriers still exist, particularly for populations with low-income and/or food insecurity, including long waitlists, limited coverage for specialized care, and geographical constraints for in-person care (86–88).

In countries with private insurance coverage, as in the United States, structural barriers to eating disorder treatment are substantial (89, 90). Being un- or under-insured, incurring high out-of-pocket expenses, and having limited financial and/or geographical access to specialty eating disorder care is unfortunately common among individuals with low income and/or food insecurity (91). Although publicly funded insurance such as Medicaid and Medicare may cover some ED care in some settings, access is still limited compared to individuals with private insurance (91–93).

Future research is critically needed to develop accessible, culturally-sensitive adaptations of existing assessment tools and frameworks for treating individuals experiencing food insecurity and eating disorders across cultures and contexts.

## Discussion

Individuals experiencing food insecurity are highly vulnerable to eating disorders. Eating disorder symptoms are detectable using brief screening measures, but additional open-ended questions may be needed to understand circumstances surrounding behaviors to help tailor interventions. Integrating mental health care alongside dietetic counseling for individuals experiencing food insecurity could help address both food insecurity and disordered eating simultaneously. Digital, self-guided interventions incorporated within existing service use may be one promising pathway forward. Finally, dietitians should be mindful of weight bias and discrimination as common experiences in this group, and provide weight-neutral care that emphasizes health behavior engagement rather than weight management.

## Data Availability

The original contributions presented in the study are included in the article/supplementary material, further inquiries can be directed to the corresponding author.
